# HPV Infection and Oral Microbiota: Interactions and Future Implications

**DOI:** 10.3390/ijms26041424

**Published:** 2025-02-08

**Authors:** Qingqing Xia, Sarah Pierson

**Affiliations:** Department of Clinical Investigation, Brooke Army Medical Center, San Antonio, TX 78234, USA; sarah.l.pierson.mil@health.mil

**Keywords:** HPV infection, oral microbiota, oral cancers, interaction, oral cancer biomarkers, probiotic treatment

## Abstract

Human papillomavirus (HPV) is a leading cause of mucosal cancers, including the increasing incidence of HPV-related head and neck cancers. The oral microbiota—a diverse community of bacteria, fungi, and viruses—play a critical role in oral and systemic health. Oral microbiota dysbiosis is increasingly linked to inflammation, immune suppression, and cancer progression. Recent studies have highlighted a complex interaction between HPV and oral microbiota, suggesting this interplay influences viral persistence, immune response and the tumor microenvironment. These interactions hold significant implications for disease progression, clinical outcomes, and therapeutic approaches. Furthermore, the oral microbiota has emerged as a promising biomarker for HPV detection and disease progress assessment. In addition, probiotic-based treatments are gaining attention as an innovative approach for preventing or treating HPV-related cancers by modulating the microbial environment. In this review, current research on the interaction between HPV and oral microbiota is provided, their clinical implications are explored, and the future potential for utilizing microbiota for diagnostic and therapeutic innovations in HPV-associated cancers is discussed.

## 1. Introduction

Human papillomavirus (HPV) is one of the most prevalent viruses associated with the development of various mucosal cancers, particularly cervical and oropharyngeal cancers. While much attention has been paid to its role in genital cancers, the increasing incidence of HPV-related head and neck cancer highlights the importance of HPV in the oral cavity [1,2]. The oral microbial flora, which includes bacteria, fungi, and virus, plays a crucial role in maintaining both oral and systemic health [3]. Dysbiosis, or imbalance, in this microbial community has been associated with excessive inflammatory reaction, immunosuppression of the host, promotion of malignant transformation, antiapoptotic activity, and secretion of carcinogens [4,5,6].

Emerging research has suggested that the interaction between HPV and the oral microbiota may play a critical role in the pathogenesis of HPV-related oral cancers, which could influence viral persistence, immune response, and the local tumor microenvironment, potentially altering disease outcomes and responses to treatment [5,7,8]. Recent studies have highlighted the complex relationship between HPV and dynamic oral microbiota, emphasizing that its balance is essential not only for oral health but also for disease progression and cancer development [9,10]. However, the role of microbial-viral interactions in the initiation, development, and progression of HPV-related cancers remains unclear.

The oral microbiota has been increasingly recognized as a biomarker for HPV-related cancers [11,12]. Understanding the interaction between HPV and oral microbiota is essential for identifying novel biomarkers for early detection, risk assessment, and therapeutic interventions. The composition of the oral microbiota has been shown to influence HPV infection persistence, and disease progression, suggesting that microbiota-based approaches could offer novel therapeutic strategies for HPV-related cancers [9]. Furthermore, manipulating the microbiota to prevent or treat HPV-related cancers is an area of growing interest [13,14,15].

In this narrative review, the aim is to provide a comprehensive overview of HPV infection and its interaction with the oral microbiota. We will explore the intricate relationship between HPV and oral microbiota, highlighting current research findings, clinical implications, and future directions in this emerging field. By advancing our understanding of these connections, we can better assess the role of oral health in HPV-related disease processes and develop targeted strategies for prevention, diagnosis, and treatment.

Approach: We conducted a narrative review by searching on PubMed, ScienceDirect, and Scopus using key words such as “HPV AND oral microbiota (microbiome), HPV AN D head-and -neck cancer (Oropharyngeal cancer, Oral cancer), oral microbiota AND treatment, oral cancer AND biomarker”. Articles published in English from 2000 to 2024 were considered. Studies were included if they discussed the role of oral microbiota in HPV-related carcinogenesis. The papers unrelated to HPV or the oral microbiome were excluded.

## 2. Overview of HPV and Oral Infections

Human papillomaviruses (HPVs) are non-enveloped, circular double-stranded DNA viruses (~8 kb) that primarily infect epithelial cells [16]. Over 200 HPV genotypes have been identified, broadly classified into low-risk and high-risk categories based on their association with benign or malignant lesions [17]. Low-risk types, such as HPV-6 and HPV-11, cause benign conditions like warts, while high-risk types, including HPV-16 and HPV-18, are linked to cancers of the cervix, anus, and the head and neck. Other high-risk types, such as HPV-31, 33, 35, and 52, show a variable frequency and geographical distribution [17,18,19,20].

**Figure 1:**
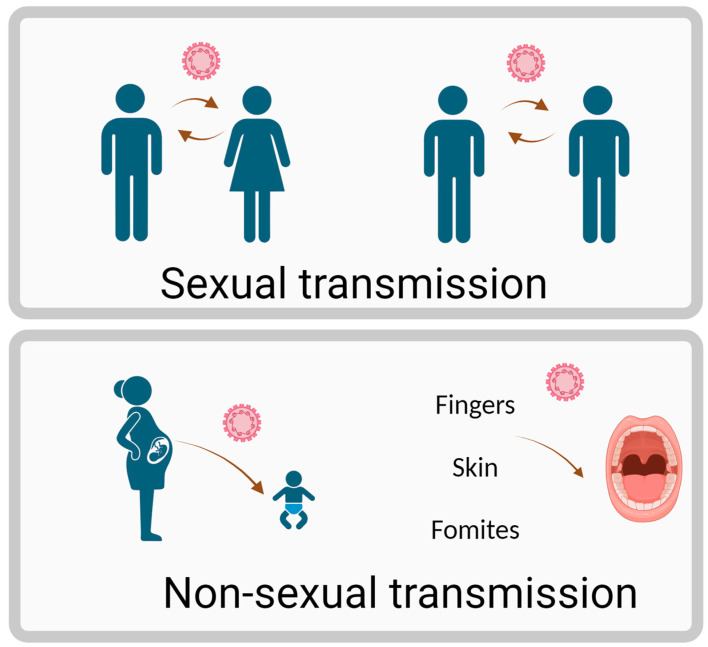
Transmission route of HPV. Figure created using Biorender.com.

HPV can be transmitted through both sexual and non-sexual routes, as illustrated in Figure 1. Oral HPV is primarily transmitted through oral–genital contact, deep kissing, and exposure to infected saliva. HPV infects the squamous cells that line the inner surface of organs, gaining access to the basal layer through micro abrasions or wounds. This infection can lead to malignancies, such as head and neck cancers in both sexes [21,22,23]. While approximately 90% of HPV infections are cleared by the innate and adaptive immune systems within 1–2 years, some infections persist due to the virus’s immune evasion strategies. These include inhibiting antigen presentation, modulating host cell apoptosis, disrupting DNA methylation, and silencing tumor suppressor genes [24,25,26]. Persistent HPV infections can integrate viral DNA into the host genome, hijacking cellular machinery for replication and altering gene expression [27,28].

The prevalence of oral HPV infections is lower than that of genital HPV infections but remains a significant concern due to its association with oropharyngeal cancers. Despite declining rates of head and neck cancers overall, the incidence of HPV-positive oropharyngeal cancers has risen, particularly among younger patients with minimal tobacco and alcohol use [29,30,31]. HPV-16 is detected in 60–80% of head and neck cancers, while HPV-18 has been identified in 34% of oral cavity squamous cell cancers and 17% of laryngeal squamous cell cancers [32]. Men have a higher prevalence of oral HPV infections (11.5% vs. 3.2%) and HPV-16 infections (1.8% vs. 0.3%) compared to women [33]. A systematic review highlighted geographic variability in HPV-positive oropharyngeal cancers, with higher rates in North America, Northern Europe, and Oceania [34]. In Europe, HPV has emerged as a growing risk factor for oropharyngeal cancer, especially among younger populations [34].

HPV status is a strong prognostic factor for oropharyngeal cancer. HPV-positive oropharyngeal squamous cell carcinomas (OPSCCs) are biologically distinct, showing increased treatment responsiveness and improved survival compared to HPV-negative tumors [35,36,37]. Some studies have shown that the presence of HPV in oropharyngeal SCC is associated with a significantly lower mortality risk and better prognosis compared to HPV-negative cases [38,39]. However, a 2022 systematic review indicated that HPV-positive oral squamous cell carcinoma (OSCC) was associated with worse overall survival (OS) and distant control (DC) compared to HPV-negative OSCC, warranting further investigation [40]. Notably, OPSCC and OSCC are two distinct subtypes of head and neck cancers that differ significantly in their anatomical origin, etiology, and clinical characteristics. OPSCC is strongly associated with high-risk HPV infection, while the correlation between OSCC and HPV remains controversial [35,41,42]. It is important to distinguish between OPSCC and OSCC when discussing HPV-related carcinogenesis and its therapeutic approaches.

Vaccination against HPV has shown substantial promise in reducing oral HPV infections. A cross-sectional study in the UK reported significantly lower oropharyngeal HPV-16 prevalence in vaccinated versus unvaccinated females (0.5% vs. 5.6%) [43]. Similarly, a U.S. study found a reduction in oral HPV-16/18/6/11 prevalence among vaccinated individuals compared to unvaccinated ones (0.11% vs. 1.61%) [44]. Vaccine efficacy appears higher among younger individuals (≤18 years: 59% effectiveness) compared to those vaccinated at older ages (≥18 years: 18% effectiveness) [45]. These findings highlight the critical role of vaccination in preventing HPV-related oral diseases and cancers.

## 3. The Oral Microbiota

The human oral microbial ecosystem includes over 700 bacterial species, of which 58% are officially named, 16% are unnamed but cultivated, and 26% are uncultivated phylotypes [46]. Beyond bacteria, the oral cavity hosts archaea, fungi, and viruses [47]. As the body’s second most diverse microbiota [48], the oral microbiome inhabits various surfaces, including the tongue, teeth, gums, and cheeks, playing a crucial role in filtering, processing, and defending against foreign elements before they reach internal systems [47,49].

Different oral niches are dominated by specific microbial species. For example, *Streptococcus* species are abundant across habitats, while *Haemophilus* dominates the buccal mucosa, *Actinomyces* the supragingival plaque, and *Prevotella* the subgingival plaque and mucosal surfaces [50,51].

The oral microbiota is vital for maintaining oral and systemic health through several key functions, as follows: (1) Pathogen Protection: commensal bacteria prevent pathogenic overgrowth by competing for nutrients and attachment sites on mucosal surfaces; (2) Immune Modulation: microbes engage with the host’s immune system to prime defenses and maintain immune tolerance; (3) Metabolic Support: oral bacteria help break down dietary components, aiding carbohydrate digestion and producing metabolic byproducts [52].

The composition of the oral microbiome is influenced by intrinsic factors (e.g., age, genetics) and extrinsic factors (e.g., diet, hygiene, tobacco, and alcohol use). Unlike other microbiota, such as the gut or skin microbiomes, the oral microbiota is distinct due to its diverse niches, constant exposure to external factors, interaction with saliva, and role in biofilm formation [52,53]. This dynamic interaction among bacteria, fungi, and viruses highlights the importance of maintaining microbial balance to prevent disease.

An imbalance in the oral microbiota, or dysbiosis, contributes to both oral and systemic diseases. In the oral cavity, bacterial dysbiosis is linked to conditions such as dental caries, periodontal disease, aphthous stomatitis, and endodontic abscesses. Furthermore, oral bacteria have been implicated in systemic diseases, including bacterial endocarditis, aspiration pneumonia, osteomyelitis, rheumatoid arthritis, cardiovascular disease, and Alzheimer’s disease [54,55,56,57]. Notably, periodontitis and HPV infection have been associated with an increased risk of OPSCCs [58]. Shin et al. highlighted that several publications have suggested the oral microbiome contribute to the etiology of different types of cancers due to their ability to alter the community composition and induce inflammatory reactions, DNA damage, apoptosis, and altered metabolism [59]. The study showed that HPV infection and cervical cancer impact not only the vaginal but also the oral microenvironments, and the oral microbial diversity exhibited an inverse pattern to that of the vaginal microbiome, indicating a unique relationship [60].

Microorganisms can colonize biotic and abiotic surfaces, including teeth, soft tissues, dental implants, and restorative materials. As microorganisms accumulate, they form structures known as biofilms, complex microbial communities enmeshed in a matrix containing extracellular polymeric substances [61], which protect the microbial community from environmental stressors, antimicrobial agents, and host immune responses [62,63]. These biofilms are primarily composed of bacteria, but can also include fungi, viruses, and archaea. In a healthy oral microbiome, biofilms play a protective role, helping to maintain the microbial balance and defend against pathogenic species. However, when dysbiosis occurs, pathogenic bacteria dominate, leading to chronic inflammation and a disruption in the biofilm’s protective functions. Pathogenic biofilms are implicated in several oral diseases, including periodontal disease, dental caries, and infections [64].

## 4. HPV and Oral Microbiota Interaction

The interaction between the oral microbiota and HPV infection is an emerging area of research, given the significant role oral microorganisms play in both maintaining oral and systemic health. HPV infection, particularly with high-risk types like HPV16, is a well-established risk factor for OPSCCs, with 40–80% of OPSCC cases linked to this virus [41,65,66,67]. However, the presence of HPV alone is insufficient for cancer development. Additional factors, such as epithelial surface integrity, mucosal secretions, immune regulation, and the surrounding microbial environment, influence how HPV infection progresses and how the immune system responds [68,69].

Recent studies have suggested that the oral microbiota may play a crucial role in HPV persistence and its associated cancer risks. Chronic oral infections, such as periodontal disease caused by pathogens such as *Porphyromonas gingivalis*, *Fusobacterium nucleatum*, and *Treponema denticola*, create a pro-inflammatory environment in the oral cavity [70]. This chronic inflammation can impair local immune responses, allowing HPV to evade detection and persist longer than in a healthy environment. Additionally, pathogens like *P. gingivalis* and *F. nucleatum* further exacerbate the issue by interfering with host immune cells, such as macrophages and dendritic cells, further facilitating HPV persistence, replication, and malignant transformation [71,72]. A prospective nested case-control study has demonstrated that a greater abundance of oral commensal bacteria in the genera *Corynebacterium* and *Kingella* were associated with a decreased risk for HNSCC, with potential implications for cancer prevention [73].

Notably, pathogenic oral bacteria may secrete certain carcinogenic metabolites that contribute to the carcinogenesis process under dysbiosis. For example, the oral bacterial species *S. salivarius*, *S. mitis*, and *S. bovis*, contain nitrate and nitrite reductases, capable of reducing the nitrate secreted in the oral cavity as a salivary component to nitrite and NO [74], carcinogenic metabolites which are associated with oral carcinogenesis [74,75]. Since *P. aeruginosa* can catalyze the reduction of nitrite to NO by cd1 nitrite reductase, a 2022 study posited that *P. aeruginosa* increases the concentration of NO in oral cavity by converting salivary nitrite to NO [76,77]. HPV infections can also exist in a latent state, where the virus is present but is not actively replicating. Under conditions of immunosuppression or microbiota dysbiosis, HPV may reactivate, leading to viral replication, shedding, and increased risk of cancer [78,79]. Chronic inflammation, driven by the presence of pro-inflammatory anaerobic bacteria, such as *Prevotella* spp., *Porphyromonas* spp., and *Fusobacerium* spp., leads to the release of inflammatory cytokines, such as interleukin (IL)-1, IL-6, tumor necrosis factor alpha (TNF-α), and interferon gamma (IFN-γ). These cytokines activate protein kinase-mediated signaling pathways, resulting in the formation of reactive oxygen species (ROS) that are associated with invasive and aggressive tumor phenotypes and tumor migration [5,6,80,81]. In one study using a 4NQO-induced mouse model of oral cancer, the inoculation of *P. gingivalis* promoted tumor progression by invading precancerous lesions and recruiting the myeloid-derived suppressor cells via the expression of chemokines such as C-C motif ligand 2 (CCL2) and chemokine (C-X-C motif) ligand 2 (CXCL2), and cytokines such as IL-6 and IL-8 [82]. Another study showed that *Rothia mucilaginosa* can generate acetaldehyde (ACH) from ethanol in vitro and induce oxidative stress in oral keratinocytes [83]. Another study suggested that dysbiosis can be dangerous due to its potential to generate ACH not only from the consumption of alcohol but also from food that does not contain alcohol [84]. Lastly, persistent inflammation depletes antioxidants, increasing oxidative damage and genetic mutation, thereby disrupting cell cycle regulation and promoting malignancy. Studies have confirmed elevated cytokine levels and reduced antioxidant defenses in patients with persistent HPV infection [80,85].

Furthermore, certain microbial profiles have been linked to HPV infection across different sites of the body. For instance, an increased abundance of *Fusobacterium* species has been associated with HPV-related cancers in the oral cavity, vagina, and anus [29,86,87]. These microbial species may not only influence HPV infection but also patient survival. As an example, *Pseudomonas viridiflava* has been associated with improved survival, while *Yersinia pseudotuberculosis* has been linked to worsened survival in head and neck cancer patients [8]. Moreover, a study has shown that the response of tumors to radiation can be regulated by the microbiome and that both the bacterial and fungal constituents of the gut microbiota impact antitumor immunity. Certain bacterial species can affect treatment response [88], which may help inform therapeutic strategies aimed at modifying the microbiome to enhance cancer treatment efficiency.

Another critical factor in the HPV–microbiota interaction is the formation of biofilms. Pathogenic biofilms, particularly those associated with periodontal disease, create a protective niche that shields HPV from immune surveillance, potentially allowing the virus to evade detection and integrate into the host genome—a key step in HPV-driven carcinogenesis [89]. Although direct evidence of HPV integration within biofilm structures is limited, biofilms likely play a role in HPV persistence by fostering immune evasion.

HPV infections can also exist in a latent state, where the virus is present but is not actively replicating. Under conditions of immunosuppression or microbiota dysbiosis, HPV may reactivate, leading to viral replication, shedding, and increased risk of cancer [78,79]. Chronic inflammation, driven by the presence of pro-inflammatory bacteria, leads to the release of proinflammatory cytokines, such as interleukin (IL)-1, IL-6, tumor necrosis factor alpha (TNF-α), and interferon gamma (IFN-γ), which activate protein kinase-mediated signaling pathways, resulting in the formation of reactive oxygen species (ROS) [80,81]. In addition, persistent inflammation depletes antioxidants, increasing oxidative damage and genetic mutations, thereby disrupting cell cycle regulation and promoting malignancy. Studies have confirmed elevated cytokine levels and reduced antioxidant defenses in patients with persistent HPV infection [80,85].

The role of fungi and other viruses in HPV infections in the oral cavity also warrants attention. *Candida albicans*, the predominant fungal species in the oral cavity, are often elevated in individuals with compromised immunity, such as HIV patients, diabetes patients, infants, and elderly populations [90]. It produces the cytolytic toxin candida lysin, leading to epithelial barrier lesions and making it easier for HPV to both infect and persist in basal epithelial cells [91]. While direct evidence of fungal involvement in HPV-driven malignancies is limited, *Candida* infections are known to produce carcinogens such as nitrosamine and promote the development of carcinoma [92]. Co-infection with other viruses, such as the Epstein–Barr virus (EBV) and herpes simplex virus (HSV), may further exacerbate HPV-related diseases. EBV is a similar virus that causes cellular transformation and chronic inflammation and may support HPV persistence [93]. In a study using an in vitro model that mimics the setting of co-infection (overexpression of E6 and E7 simulating HPV integration followed by EBV infection), the cancerous cell line FaDu and the normal cell line NOK demonstrated a significant increase in invasion in the absence of any effect on proliferation [94]. 

The synergistic effects of chronic inflammation and co-infections with bacteria, fungi, and viruses in the oral cavity create a persistent inflammatory environment that supports HPV persistence, reactivation, and oncogenesis. The evidence suggests that individuals with periodontal disease or other chronic oral infections may be at a higher risk for developing HPV-related cancers, emphasizing the need to maintain a balanced oral microbiota to reduce cancer risk [95,96].

## 5. Oral Microbiota as a Biomarker for HPV-Related Cancers

Oral squamous cell carcinoma (OSCC) is the most common type of oral cancer, accounting for approximately 90% of cases [97]. The five-year survival rate for OSCC is around 30–41% in advanced stages, but it exceeds 85% when detected early [98,99]. Unfortunately, nearly half of OSCC cases are diagnosed at later stages, making early detection critical for improving patient outcomes [100]. Reliable biomarkers for detecting and predicting the progression of HPV-related oral cancers, including OSCC, are urgently needed.

Traditionally, biomarkers such as HPV DNA, p16 expression, and antibodies against HPV oncogenes (e.g., E6 and E7) have been used to assess the risk of HPV-driven malignancies [101,102]. However, the recent evidence has suggested that changes in the oral microbial composition—particularly patterns of dysbiosis—may provide valuable insights into the risk and progression of HPV-related oral cancers, offering a non-invasive alternative to traditional biomarkers. Saliva has emerged as a promising medium for detecting both HPV DNA and microbial signatures. Salivary samples can provide a comprehensive overview of the oral microbial ecosystem and its interactions with HPV, offering a snapshot of the dynamic changes occurring within the oral cavity [10,11]. Advances in sequencing technologies and metagenomic analyses have made it possible to identify even subtle shifts in microbial composition, allowing for the detection of early dysbiosis associated with HPV-related malignancies [102,103].

Although many studies have examined the microbiome in the oral cavity and oropharyngeal cancers, most do not specify HPV positivity. In a study of oral cavity cancers with unknown HPV status, the bacterial composition of tumor sites was significantly different from that of contralateral normal mucosa samples [104]. Furthermore, patients with detectable oral HPV or HPV-positive OPSCC exhibit significant shifts in their oral microbiomes compared to patients with HPV-negative counterparts [105].

Healthy individuals typically maintain a diverse and balanced oral microbiota dominated by commensal species that promote oral health. In contrast, individuals with HPV-related oropharyngeal cancers often experience oral dysbiosis, characterized by an overrepresentation of pathogenic species and a reduction in beneficial commensals. Patients with oral HPV exhibit similar alpha diversity in their oral microbiomes to healthy individuals but show distinct differences in beta-diversity [9,106]. Specifically, HPV-positive patients demonstrate an increased abundance of *Actinomycetaceae*, *Prevotellaceae*, *Veillonellaceae*, *Campylobacteraceae*, and *Bacteroidetes*, while species such as *Gemellaceae*, *Neisseria*, and *Lactobacillus* are less abundant [9,106].

In HPV-positive cancer patients, members of the genera *Actinomyces*, *Granulicatella*, *Oribacterium*, and *Campylobacter*, as well as the species *Veillonella dispar*, *Rothia mucilaginosa*, and *Haemophilus parainfluenzae*, are significantly increased, while *Streptococcus anginosus*, *Peptoniphilus*, and *Mycoplasma* are significantly decreased [107]. Furthermore, Mougeot et al. identified higher relative abundances of *Alloprevotella tannerae*, *Fusobacterium periodonticum*, *Haemophilus pittaniae*, and *Leptotrichia* spp. in HPV-positive head and neck cancer patients compared to HPV-negative patients [108]. Additionally, studies have found that patients with HPV-positive oropharyngeal cancers often show increased abundance of bacterial taxa such as *Fusobacterium nucleatum*, *Porphyromonas*, and *Treponema denticola*, which are also associated with periodontal disease [109]. The recent research has demonstrated that significant shifts in the composition of the oral microbiota occur by disease stage and after chemoradiotherapy in HPV-positive oropharyngeal squamous cell carcinoma patients [86], indicating oral microbiota could play a role in disease progression, response to treatment, or recovery, highlighting its potential as a therapeutic target or biomarker.

The shift in oral microbial communities in HPV-related cancers may contribute to carcinogenesis through several mechanisms. Pathogenic bacteria associated with oral dysbiosis can promote chronic inflammation, a well-established factor in cancer development. Certain bacterial species, such as *Fusobacterium nucleatum*, have been shown to modulate the immune response, potentially facilitating HPV persistence and promoting epithelial cell transformation. In a mouse model of oral tumorigenesis, co-infection with *Porphyromonas gingivalis* and *Fusobacterium nucleatum* significantly enhanced the severity of tongue tumors and increased IL-6 levels in the tongue epithelium [110]. Furthermore, bacterial metabolites may influence the host’s immune surveillance or directly interact with HPV to enhance viral replication. These microbial-induced changes in the local immune environment could create a favorable niche for HPV infection and persistence, critical steps in the development of oral cancers.

The identification of specific microbial signatures linked to cancer progression could aid in risk stratification, allowing clinicians to monitor individuals with high-risk HPV infection more closely. Longitudinal studies that track microbiota changes in HPV-positive individuals could help define microbiota profiles associated with either viral clearance or progression to malignancy. These microbial signatures could be incorporated into predictive models to improve early detection and intervention.

Despite the potential of the oral microbiota as a biomarker for HPV-related cancer, several challenges remain. One major hurdle is the variability in the oral microbiota among individuals, which can be influenced by factors such as diet, smoking, hygiene, and genetics [111]. These confounding variables make it difficult to establish universal microbial signatures that can be applied across diverse populations.

In the future, oral microbiota-based diagnostic tests could be integrated into clinical practice to enhance early detection strategies for HPV-related cancers. By combining traditional HPV biomarkers with microbial profiling, clinicians may be able to develop more comprehensive screening protocols that improve patient outcomes. Such testing could also be used to monitor treatment responses, as changes in the oral microbiota may reflect shifts in the immune response or cancer progression following therapy.

Additionally, more research is needed to establish causality between microbial shifts and HPV-related cancer progression. While associations between specific bacterial species and cancer have been observed, it is not yet clear whether these microbial changes are a cause or consequence of disease progression. Longitudinal studies that track microbial changes from the onset of HPV infection to cancer development are essential to clarify these relationships.

Finally, standardization of sampling techniques and analytical methods is necessary to ensure consistent and reproducible results across studies. As the field advances, the development of standardized protocols for microbiota sampling, sequencing, and analysis will be crucial for translating research findings into clinical applications.

## 6. Therapeutic Implications and Future Directions

In most oral carcinoma cases, the main treatment method is surgical removal. In advanced cases, postoperative radiotherapy, chemotherapy, oncogene-targeted therapy, and immunotherapy may be administered [112]. However, these treatments can cause alteration in oral microflora and lead to oral mucositis, oral candidiasis, and hyposalivation [113,114]. Another important role of the oral microbiome is modulating the immune system and influencing the effectiveness of treatments for oral cancers. Overgrowth of opportunistic pathogens like Staphylococci, enteric rods, and Candida sp. tend to increase in prevalence after radiotherapy with or without chemotherapy [115]. In a study from China, mutant streptococci were not isolated in radiotherapy patients while lactobacilli, *S. mitis*, and *S. salivarius* mutation increased significantly following radiotherapy [116]. Some studies have shown that modulating or manipulating the oral microbiome can help alleviate the side effects caused by cancer therapies. A randomized, double-blind, placebo-controlled trial showed that probiotics can significantly enhance the immune response of patients and reduce the severity of oral mucositis induced by chemoradiotherapy through modification of gut microbiota [117]. *Lactobacillus brevis* CD2 lozenges have been shown to reduce the severity and incidence of radio/chemotherapy-induced mucositis in head and neck cancer patients, thereby increasing the likelihood of anticancer treatment completion [118]. Studies have pointed out microbial species which can be categorized as pro-tumorigenic and anti-tumorigenic [107,119,120]. One study indicated that the presence of *Luteibacter*, *Flammeovirgo*, and *Lachnoclostridium* was correlated with total T-cell receptor reads, number of clones, leukocytes, and CD8+ T-cell infiltration suggesting their potential influence on the tumor microenvironment and immunotherapy response regulation [37]. It was proposed by Sivan et al. that oral administration of *Bifidobacterium*, a particular taxon of microbial commensals, improved melanoma control to the same degree as programmed cell death protein 1 ligand 1 (PD-L1) specific antibody therapy (checkpoint blockade), and combination treatment nearly abolished tumor outgrowth in a mouse model [121].

The relationship between the oral microbiota and HPV infection offers promising therapeutic opportunities. A study analyzing vaginal flora in 26 HPV patients found significantly lower bacterial diversity in those who cleared HPV within a year compared to those with persistent infection [122], suggesting that a healthy microbiota aids HPV clearance. Although oral and vaginal microbiota differ, it is reasonable to hypothesize that a healthy oral microbiota could similarly support oral HPV clearance. Beyond influencing infection dynamics, the oral microbiota plays a critical role in immunity, making microbial balance restoration a potential approach for reducing HPV-related cancer risk. Thus, restoring an imbalanced oral microbiota may be the fundamental key for preventing HPV persistence and progression to malignancy.

Current studies on considering probiotics as an HPV treatment have primarily focused on cervical rather than oral cancer. While data on oral HPV are limited, some studies have shown promising probiotic effects on cervical cancer cell lines (Table 1). Clinical trials have also explored probiotics for HPV-related infections. A prospective study of 54 HPV-positive patients with low-grade squamous intraepithelial lesions showed that daily consumption of *Lactobacillus casei Shirota* (Yakult) doubled the chance of clearing cytological abnormalities (*p* = 0.05). HPV clearance occurred in 19% of controls versus 29% of probiotic users (*p* = 0.41), indicating potential benefits [123]. Another study with 100 women found that vaginal *L. crispatus chen-01* transplantation significantly reduced HPV viral load, improved clearance rates, and ameliorated vaginal inflammation without notable side effects [124]. Conversely, a randomized, double-blinded trial found no significant difference in high-risk HPV clearance between a probiotic group (*Lactobacillus rhamnosus* GR-1 and *Lactobacillus reuteri* RC-14) and a placebo group [125]. However, the bacterial concentration used in this study was lower than other studies, the strains that were used in this study were also different from the other study, indicating dosing, strain, and duration may all play important roles in viral clearance. A summary of the probiotics used for HPV-related infections is presented in Table 2.

While most current studies focus on cervical HPV infections, research on the role of probiotics in oral HPV remains underexplored. Preliminary studies in other areas of the body, such as the vaginal microbiota, have shown that probiotics can be effective in reducing viral infections and improve immune function. These findings suggested that a similar approach could be applied to the oral cavity. Given the differences between oral and vaginal microbiota, understanding how probiotics influence oral HPV clearance is crucial for developing future, targeted therapies. Future research should prioritize clinical trials investigating the effects of probiotics on oral HPV infections, focusing on identifying specific strains and treatment durations that optimize clearance rates. Additionally, studies should aim to determine the ideal composition of a “healthy” oral microbiota in the context of HPV. Despite promising results, current findings are limited by variability in probiotic strains, dosages, and study designs, underscoring the need for standardized approaches and larger sample sizes.

Since microbial dysbiosis may serve as a bridge between periodontal disease and oral cancer, another potential therapeutic avenue to restore oral dysbiosis is the direct targeting of pathogenic bacteria associated with HPV persistence. Antimicrobial therapies, such as antibiotics or antimicrobial peptides, could be used to eliminate or reduce the abundance of specific bacterial species that promote chronic inflammation and create a conducive environment for HPV. However, this approach requires careful consideration, as broad-spectrum antibiotics could disrupt the overall balance of the oral microbiota, leading to unintended consequences. A more targeted approach could involve the use of narrow-spectrum antimicrobial peptides or bacteriophages. Notably, the functional peptides derived from bacteriophages offer the benefits of targeting cancer cells with high specificity while causing minimal immune response in humans. A study revealed that oral vaccination with bacteriophage MS2-L2 virus-like particles (VLPs) could provide protection against oral infections caused by multiple HPV types linked to head and neck cancers [126], highlighting the potential of phage-based vaccines in combating oral cancers. By selectively targeting pathogenic bacteria while preserving beneficial commensals, these therapies could reduce inflammation and improve immune responses without causing widespread disruption of the microbiota. Integrating HPV vaccination and phage vaccination into preventive strategies for oral dysbiosis and HPV infections could significantly enhance control measures. HPV vaccination would reduce the incidence of HPV-related oral cancers, while phage vaccination could specifically target oral pathogens associated with HPV persistence.

As the research on HPV and the oral microbiota continues to evolve, the future of therapeutic interventions is likely to focus on personalized medicine. Personalized medicine approaches could involve using the oral microbiota as both a diagnostic tool and a therapeutic target. By identifying specific microbial profiles associated with increased cancer risk, clinicians could tailor treatment strategies to reduce pathogenic bacterial populations, restore microbial balance, and modulate immune responses. Advances in microbiome research, combined with genetic and immune profiling, may allow for the development of individualized treatment plans that take into account each patient’s unique microbial and immune landscape. Together with personalized microbiota interventions, these tools could provide a multi-layered, individualized approach to prevent and treat oral dysbiosis and HPV infections, improving overall oral health outcomes. This combined strategy holds great promise for the future of preventive healthcare in oral diseases.

**Table 1:** Summary of probiotic effects on HPV-related cancer cells.

**Bacteria/Probiotics**	**Cell Line**	**Outcome**	**References**
Milk-isolated *L. casei* and *L. paracasei*	HeLa	Upregulating the expression of apoptotic genes	[127]
Vagina-isolated *L. gasseri*	HeLa	Inflammation and proliferation were reduced, and apoptosis was increased	[128]
Vagina-isolated *L. plantarum*	HeLa	Suppression of proliferation and induction of apoptosis	[129]
Supernatants of *L. rhamnosus* and *L. crispatus*	HeLa	Inhibited cell proliferation and metastasis	[130]
Supernatants of *L. rhamnosus* and *L. crispatus*	HeLa	Decrease expression of HPV oncogenes	[131]
* Lacticaseibacillus casei * LH23	HeLa	suppress the proliferation and induced the apoptosis of cervical cancer cells	[132]
Bifdobacterium adolescentis SPM1005-A	SiHa	Inhibited E6 and E7 oncogenes	[133]
supernatants of *L. crispatus*, *L. jensenii*, and *L. gasseri*	CasKi	inhibitory effects on the viability of cervical cancer cells via regulation of HPV oncogenes and cell cycle-related genes	[134]
Twelve standard *Lactobacillus*	HeLa and SiHa	Reduce tumor invasion and metastasis	[135]

**Table 2:** A summary of studies exploring the use of probiotics for HPV-related infections.

Sample Size	Bacterial Strain	Condition	Intervention	Outcome	References
N = 160	*L. crispatus* M247	ASCUS * or LSIL * HPV+	Oral administration (no fewer than 20 billion) for 12 months	higher percentage of clearance of PAP-smear abnormalities in patients who took oral *Lactobacillus crispatus* M247 than in the control group	[136]
N = 35	*L. crispatus* M247	ASCUS or LSIL or NILM * HPV+	Oral administration (no fewer than 20 billion) for 3 months	reduction of approximately 70% in HPV positivity and a significant change in CST status with 94% of women	[137]
N = 54	* L. casei * Shirota	LSIL HPV+	Oral administration (no fewer than 20 billion) for 6 months	Probiotic users had a twice as high chance of clearance of cytological abnormalities	[123]
N = 121	* L. rhamnosus * and *L. reuteri*	High-risk HPV	Oral administration (no fewer than 5.4 billion) discontinued until negative HPV testing	No significant influence on HR-HPV clearance, but may have decreased the rates of mildly abnormal and unsatisfactory cervical smears	[125]
N = 91	*L. crispatus* chen01	High-risk HPV	Intravaginal 1 × 10^9^ CFU per capsule for 5 months	Significantly reduced viral load of HPV, ameliorated HPV clearance rate, and improved vaginal inflammation state	[124]
N = 117	* L. rhamnosus * BMX 54	C1N1 *, HPV+	Intravaginal 1 × 10^4^ CFU per tablet for 3 or 6 months	Long term user doubled the chance of resolving HPV-related cytological anomalies compared to short term user	[138]

* ASCUS: atypical squamous cells of undetermined significance; LSIL: low-grade squamous intraepithelial lesion; NILM: negative for intraepithelial lesion or malignancy; C1N1: cervical intraepithelial neoplasia grade 1.

## 7. Conclusions

By synthesizing the current findings, in this review, the importance of understanding how oral microbiota interaction develops in cancer pathogenesis has been underscored and the need for further studies to establish new strategies for prevention, diagnosis, and treatment of HPV-associated oral diseases has been highlighted. While much progress has been made, longitudinal studies, large-scale clinical trials, and in-depth microbiome analysis will be needed to identify potential biomarkers, therapeutic targets, and strategies for intervention. In conclusion, the interaction between HPV and oral microbiota represents a promising area of research that could revolutionize the prevention, diagnosis, and treatment of HPV-related oral cancers.
